# The effect of pentadecapeptide BPC 157 on hippocampal ischemia/reperfusion injuries in rats

**DOI:** 10.1002/brb3.1726

**Published:** 2020-06-18

**Authors:** Jakša Vukojević, Borna Vrdoljak, Dominik Malekinušić, Marko Siroglavić, Marija Milavić, Danijela Kolenc, Alenka Boban Blagaić, Lovorka Batelja, Domagoj Drmić, Sven Seiverth, Predrag Sikirić

**Affiliations:** ^1^ Department of Pharmacology Medical School University of Zagreb Zagreb Croatia; ^2^ Department of Pathology Medical School University of Zagreb Zagreb Croatia

**Keywords:** BPC 157, cytoprotection, hippocampus, ischemia, ischemia injuries, nitric oxide, peptide, reperfusion

## Abstract

**Background and purpose:**

We focused on the, yet undescribed, therapy effect of the stable gastric pentadecapeptide BPC 157 in hippocampal ischemia/reperfusion injuries, after bilateral clamping of the common carotid arteries in rats. The background is the proven therapy effect of BPC 157 in ischemia/reperfusion injuries in different tissues. Furthermore, there is the subsequent oxidative stress counteraction, particularly when given during reperfusion. The recovering effect it has on occluded vessels, results with activation of the alternative pathways, bypassing the occlusion in deep vein thrombosis. Finally, the BPC 157 therapy benefits with its proposed role as a novel mediator of Roberts’ cytoprotection and bidirectional effects in the gut‐brain axis.

**Materials and Methods:**

Male Wistar rats underwent bilateral clamping of the common carotid arteries for a 20‐min period. At 30 s thereafter, we applied medication (BPC 157 10 µg/kg; or saline) as a 1 ml bath directly to the operated area, that is, trigonum caroticum. We documented, in reperfusion, the resolution of the neuronal damages sustained in the brain, resolution of the damages reflected in memory, locomotion, and coordination disturbances, with the presentation of the particular genes expression in hippocampal tissues.

**Results:**

In the operated rats, at 24 and 72 hr of the reperfusion, the therapy counteracted both early and delayed neural hippocampal damage, achieving full functional recovery (Morris water maze test, inclined beam‐walking test, lateral push test). mRNA expression studies at 1 and 24 hr, provided strongly elevated (*Egr1, Akt1, Kras, Src, Foxo, Srf, Vegfr2, Nos3, and Nos1*) and decreased (*Nos2, Nfkb*) gene expression (*Mapk1* not activated), as a way how BPC 157 may act.

**Conclusion:**

Together, these findings suggest that these beneficial BPC 157 effects may provide a novel therapeutic solution for stroke.

## Graphical abstract

Pentadecapeptide BPC 157 is a novel mediator of Roberts cytoprotection and has bidirectional effects on the gut‐brain axis. BPC 157 effectively ameliorated ischemia/reperfusion injuries and as such is a possible new therapeutic solution in ischemic/reperfusion injuries.

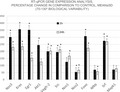

## 1 INTRODUCTION

Stroke remains one of the leading causes of death and long‐term disability, and the urgent quest for an effective therapeutic solution is evidenced by numerous failed clinical trials (Holloway & Gavins, 2016) but also in novel ways of trying to treat the disease (Amani, Habibey, et al., 2019; Amani, Kazerooni, Hassanpoor, Akbarzadeh, & Pazoki‐Toroudi, 2019). Although ischemia is the main culprit in stroke‐related injuries, another crucial part of the stroke is reperfusion, an event taking place after the ischemic area is resupplied with blood (Chamorro, Dirnagl, Urra, & Planas, 2016; Jayaraj, Azimullah, Beiram, Jalal, & Rosenberg, 2019). It is vital to re‐establish blood flow, but this act also contributes to neuronal injury by activating an immunological response and leading to endotoxicity, neuroinflammation, and oxidative and nitrosative stress, thereby worsening the outcome of ischemia (Chamorro et al., 2016; Jayaraj et al., 2019).

Therefore, we focused on the yet undescribed therapeutic effect of the stable gastric pentadecapeptide BPC 157 (Kang et al., 2018; Seiwerth et al., 2014, 2018; Sikiric et al., 2010, 2011, 2012, 2013, 2014, 2016, 2017, 2018) in reperfusion of ischemia/reperfusion stroke injuries after bilateral clamping of the common carotid artery in rats. With the BPC 157 therapy administered during reperfusion, we would achieve the resolution of damage sustained in the brain, in particular in hippocampal regions CA1‐4 similar to damage reflected in memory, locomotion, and coordination disturbances. Likewise, this notion will be further supported by the detection of expression of particular genes (*Vegfr2, Src, Nos2, Nos1, Nos3, Akt1, Kras, Mapk1, Srf, Foxo1, Nfkb1,* and *Egr1*) in hippocampal tissue.

The possible therapeutic effect may be due to the specific beneficial effect of BPC 157 in the ischemia/reperfusion injuries and oxidative stress counteraction, particularly when given during reperfusion (Amic et al., 2018; Drmic et al., 2018; Duzel et al., 2017; Sever et al., 2019; Vukojevic et al., 2018). Likewise, there is the recovering effect it has on occluded vessels and bypassing the occlusion (Amic et al., 2018; Drmic et al., 2018; Duzel et al., 2017; Sever et al., 2019; Vukojevic et al., 2018). As a final point, BPC 157 therapy ameliorates deep vein thrombosis, inferior caval vein occlusion, colitis ischemia/reperfusion, duodenal venous congestion, and cecum perforation (Amic et al., 2018; Drmic et al., 2018; Duzel et al., 2017; Sever et al., 2019; Vukojevic et al., 2018). It was recently reported that after induction of liver cirrhosis due to both bile duct ligation (Sever et al., 2019) and portal hypertension, pre‐existing portal hypertension was prevented and reversed to normal values (Sever et al., 2019) due to BPC 157 therapy. The important point may be that BPC 157 is an original anti‐ulcer agent that is native and stable in human gastric juice (for more than 24 hr) (Sikiric et al., 2010). It was tested in trials for ulcerative colitis and now multiple sclerosis, and it has a very safe profile (lethal dose (LD1) not achieved). Furthermore, as an important conceptual and practical point, BPC 157 is thought to be a novel mediator of Robert's cytoprotection, the process that accompanies epithelium and endothelium protection, maintains gastrointestinal mucosal integrity, and has an organoprotection effect in preserving the integrity of other organ systems (Kang et al., 2018; Seiwerth et al., 2014, 2018; Sikiric et al., 2010, 2011, 2012, 2013, 2014, 2016, 2017, 2018). Consequently, BPC 157 plays an important role in the gut‐brain and brain‐gut axis due to its important beneficial effects on various CNS (central nervous system) disorders (Kang et al., 2018; Seiwerth et al., 2014, 2018; Sikiric et al., 2010, 2011, 2012, 2013, 2014, 2016, 2017, 2018). Illustratively, BPC 157 inhibits amphetamine‐induced stereotypy and haloperidol‐induced catalepsy disturbances (Jelovac et al., 1998, 1999; Sikiric et al., 2002) and mainly interacts with the NO system (for review see, that is, (Sikiric et al., 2014, 2016)). Conceptually, the particular beneficial effect is that BPC 157 affects these disturbances when given peripherally. Namely, BPC 157 induces the release of serotonin in specific brain nigrostriatal regions and influences serotonergic and dopaminergic systems (Tohyama, Sikiric, & Diksic, 2004). Consequently, the beneficial effects appear against the specifically (over)stimulated or damaged dopaminergic, serotonergic, GABAergic, and opioid systems (Sikiric et al., 2016). Additionally, BPC 157 counteracts several encephalopathies after overdose of NSAIDs (Drmic et al., 2017; Ilic et al., 2009, 2010; Ilic, Drmic, Franjic, et al., 2011; Ilic, Drmic, Zarkovic, et al., 2011; Lojo et al., 2016), insulin (Ilic et al., 2009) or cuprizone, which is a neurotoxin that induces multiple sclerosis‐like damage in rats (Klicek et al., 2013). Likewise, in brain‐trauma studies, BPC 157 counteracts brain lesions and markedly improves consciousness in injured mice (Tudor et al., 2010). Furthermore, BPC 157 significantly improves nerve healing and function after sciatic nerve transection with and without anastomosis (Gjurasin et al., 2010). Very recently, BPC 157 markedly enhanced the survival of cultured enteric neurons but did not influence the proliferation of cultured enteric glial cells (Wang et al., 2019). BPC 157 healing is known to function through several molecular pathways (Chang, Tsai, Hsu, & Pang, 2014; Chang, Tsai, Lin, Hsu, & Pang, 2011; Hsieh et al., 2017; Huang et al., 2015; Kang et al., 2018; Tkalcevic et al., 2007; Vukojevic et al., 2018). Illustratively, along with the counteracted tumor cachexia (Kang et al., 2018), muscle wasting and changes in the expression of *Foxo3, Akt1, Mtor,* and *Gsk3b*, BPC 157 counteracts increased proinflammatory cytokines such as IL‐6 and TNF‐α (Kang et al., 2018). Thereby, we tested BPC 157 in rats after bilateral clamping of the common carotid arteries.

## 2 MATERIALS AND METHODS

### 2.1 Animals

Study protocols were conducted in male Albino Wistar rats that had a body weight 200–250 g, were 12 weeks old and were bred in‐house at the animal facility of the Department of Pharmacology‐ School of Medicine, Zagreb, Croatia. This is an animal facility registered with the Directorate of Veterinary (Reg. No: HR‐POK‐007). Laboratory rats were acclimated for 5 days and randomly assigned to their respective treatment group (at least eight rats for each experimental group, depending on the method that was evaluated). Laboratory animals were housed in PC cages in conventional laboratory conditions at a temperature of 20°C–24°C, a relative humidity of 40%–70% and noise level of 60 DCB. Each cage was identified with the date, the number of the study, group, dose, number, and sex of each animal. Fluorescent lighting provided illumination 12 hr per day. A standard good laboratory practice diet and fresh water were provided ad libitum. Animal care complied with SOPs of the Pharmacology Animal Facility and the European Convention for the Protection of Vertebrate Animals used for Experimental and Other Scientific Purposes (ETS 123). Ethical principles of the study ensured compliance with European Directive 010/63/E, the Law on Amendments to Animal Protection Act (Official Gazette 37/13), the Animal Protection Act (Official Gazette 135/06), Ordinance on the protection of animals used for scientific purposes (Official Gazette 55/13), FELASA recommendations and recommendations of the Ethics Committee School of Medicine, University of Zagreb. Observers, who were blind to treatment regimens, assessed the experiments and evaluated the neurological tests.

### 2.2 Drugs

As previously described (Kang et al., 2018; Seiwerth et al., 2014, 2018; Sikiric et al., 2010, 2011, 2012, 2013, 2014, 2016, 2017, 2018), the medication without carrier or peptidase inhibitor included stable gastric pentadecapeptide BPC 157 (a partial sequence of the human gastric juice protein BPC, freely soluble in water at pH 7.0 and in saline). It was prepared as a peptide with 99% (HPLC) purity (1‐des‐Gly peptide was the main impurity; manufactured by Diagen, Ljubljana, Slovenia, GEPPPGKPADDAGLV, M.W. 1419) (in dose and application regimens as described before) (Kang et al., 2018; Seiwerth et al., 2014, 2018; Sikiric et al., 2010, 2011, 2012, 2013, 2014, 2016, 2017, 2018).

### 2.3 Surgery procedure and medication

Rats were anesthetized by intraperitoneal injection of thiopental (50 mg/kg) and diazepam (5 mg/kg). After anesthesia induction, each rat was placed in the supine position and fixed on the operating table. A midline incision of approximately two centimeters was made in the neck, and then both common carotid arteries and common jugular veins were exposed carefully by blunt dissection. After the vagus nerve was carefully separated from the carotid artery, cerebral ischemia was induced by bilateral clamping of the common carotid arteries. Bilateral clamping of the common carotid arteries was relieved at the end of the 20‐min period. Thirty seconds later, we applied medication (BPC 157 10 µg/kg; or saline as a 1 ml bath directly on the surgical area. Five minutes after that, the incision was sutured back in layers. The sutured area was cleaned with 70% ethanol and sprayed with an antiseptic solution.

### 2.4 Neurological assessment

#### 2.4.1 Morris water maze test

We examined spatial learning and memory of the rats by using a spatial version of the Morris water maze (MWM) test (Vorhees & Williams, 2006). The training period was conducted through five consecutive days before the animals underwent surgery. There was a final assessment 24 hr after surgery. In each training period, the rats received four trials (with an intertrial gap of 10 min) in which the invisible platform was placed in the same location. The trial was complete once the rat found the platform and remained on the platform for 10 s or until 60 s had elapsed. To assess spatial learning, the latency time to find the invisible platform was measured for each animal. The Day 5 latency time (seconds), which was averaged from 4 trials, to locate the hidden platform in the water maze was taken as an index of acquisition or learning and a baseline measure. The difference between training latency time and postoperative latency time (ΔT) was calculated.

#### 2.4.2 Inclined beam walk test

Locomotor capabilities were evaluated using the inclined beam‐walking test, as described previously (Gulati & Singh, 2014). Each animal was individually placed on a 110 cm long and 1.5 cm wide wooden bar that was inclined at an angle of 60° from the ground, and the rats were given 60 s to walk the beam. The motor performance of each rat was scored (0–4) before global cerebral ischemia and 24 hr after global cerebral ischemia/reperfusion, as follows: 0—completely unable to walk on the beam; 1—able to walk less than ¼ of the beam length; 2—able to walk more than ¼ but less than ½ of the beam length; 3—able to walk more than ½ but less than ¾ of the beam length; and 4—able to more than ¾ of the beam length or to walk the whole beam in 60 s.

#### 2.4.3 Lateral push test

Before stroke inducing surgery and at 24 hr after surgery, the animal was placed on a rough surface for firm grip and was evaluated for resistance to a lateral push from either side of the shoulder, as described previously (Gaur, Aggarwal, & Kumar, 2009). An animal with increased or decreased resistance to a lateral push after ischemia was assigned a + or – score, respectively (Gaur et al., 2009), and the percentage of rats showing resistance to a lateral push was recorded.

### 2.5 Histopathological analysis of the hippocampus

Whole brains were fixed in 10% neutral buffered formalin for 48 hr. After fixation, coronal sections were made through the middle part of the hippocampus. Brain slabs were dehydrated in graded ethanol (70%, 80%, 96%, and 100%) and embedded in paraffin. Paraffin blocks were cut into 4–5 μm thin sections, deparaffinated in xylene, rehydrated in graded ethanol (100%, 96%, 80%, and 70%) and stained with hematoxylin and eosin. Five images were taken of the CA1 region of the hippocampus with high power (objective ×40). The healthy neurons and "red neurons" (pathological neuronal finding indicative of acute ischemic neuronal injury and subsequent apoptosis or necrosis) were manually counted and presented as an average of five images at 24 and 72 hr after surgery.

### 2.6 RT‐qPCR mRNA measurement

After sacrificing the animals 1 and 24 hr after reperfusion, brain tissue, that is, the hippocampus, was rapidly dissected and frozen in liquid nitrogen. The hippocampal tissue was disrupted using a T10 Ultra‐turrax homogenizer (IKA Werke GmbH) followed by RNA extraction with TRIzol Reagent (Invitrogen, Thermo Fisher Scientific) according to the manufacturers’ instructions. After RNA isolation, the nucleic acid concentration was measured with a NanoDrop ND‐1000 spectrophotometer (NanoDrop Technologies). Reverse transcription was performed using a High Capacity cDNA Reverse Transcription Kit (Applied Biosystems, Thermo Fisher Scientific) with 1,000 ng of RNA following the manufacturer's instructions and using a ProFlex PCR System (Applied Biosystems, Thermo Fisher Scientific) machine. For gene expression analysis, TaqMan Gene Expression Assays (Applied Biosystems, Thermo Fisher Scientific, Massachusetts, USA) were used (Table 1, additional material). We analyzed three different endogenous controls, *Gadph, Actb,* and *Rn18s*, and several genes of interest, *Vegfr2, Src, Nos2, Nos1, Nos3, Akt1, Kras, Mapk1, Srf, Foxo1, Nfkb1,* and *Egr1*. Reactions were performed with a 7500 Real‐Time PCR System (Applied Biosystems, Thermo Fisher Scientific). The results were expressed as a fold change in comparison with control samples (expressed as percentages), and the range between 70% and 130% was considered biological variability (Maul, 2008; Vukojevic et al., 2018).

**TABLE 1:** Gene and primer table

Gene symbol	Synonyms	Gene name	TaqMan Assay ID	NCBI Reference Sequence	Amplicon length (bp)
*Actb*	Actx	Actin, beta	Rn00667869_m1	NM_031144.3	91
*Akt1*	PKB, RAC	AKT serine/threonine kinase 1	Rn00583646_m1	NM_033230.2	87
*Egr1*	NGFI‐A, Krox‐24, Zif268	Early growth response 1	Rn00561138_m1	NM_012551.2	64
*Foxo1*	Fkhr	Forkhead box O1	Rn01494868_m1	NM_001191846.2	99
*Gapdh*	Gapd	Glyceraldehyde‐3‐phosphate dehydrogenase	Rn01775763_g1	NM_017008.4	174
*Kras*	Ki‐Ras, Kras2	KRAS proto‐oncogene, GTPase	Rn00580460_m1	NM_031515.3	125
*Mapk1*	Erk2, ERT1, p42mapk	Mitogen activated protein kinase 1	Rn00671828_m1	NM_053842.1	102
*Nfkb1*	EBP‐1, NF‐kB	Nuclear factor kappa B subunit 1	Rn01399572_m1	NM_001276711.1	67
*Nos1*	nNOS, bNOS	Nitric oxide synthase 1	Rn00583793_m1	NM_052799.1	65
*Nos2*	iNos, Nos2a	Nitric oxide synthase 2	Rn00561646_m1	NM_012611.3	77
*Nos3*	eNos, cNOS	Nitric oxide synthase 3	Rn02132634_s1	NM_021838.2	117
*Rn18s*	‐	18S ribosomal RNA	Rn03928990_g1	NR_046237.1	61
*Src*	c‐Src, p60‐Src	SRC proto‐oncogene, non‐receptor tyrosine kinase	Rn00583063_m1	NM_031977.1	75
*Srf*	LOC501099	Serum response factor	Rn01757240_m1	NM_001109302.1	65
*Vegfa*	VEGF‐A, VPF	Vascular endothelial growth factor A	Rn01511601_m1	NM_001110333.2	69

Specifications for TaqMan Assays genes and primers used in the experiment (Rat Genome Database and Alliance of Genome Resources nomenclature data).

### 2.7 Statistical analysis

Statistical analysis was performed using the Shapiro–Wilk test for distribution assessment. For parametric statistics, we used the Mann–Whitney *U* test to compare the difference between groups. Additionally, for nonparametric statistics, we used Kruskal–Wallis test to compare the two groups. All statistical tests were performed using RStudio (RStudio Team, 2015). A *p*‐value of .05 or less was considered statistically significant.

## 3 RESULTS

After bilateral carotid artery occlusion, significant reperfusion‐induced hippocampal lesions, as well as memory and motor coordination failure, regularly appeared in tested animals. These disturbances clearly indicate our therapeutic focus.

### 3.1 Neurological assessment

**FIGURE 1:**
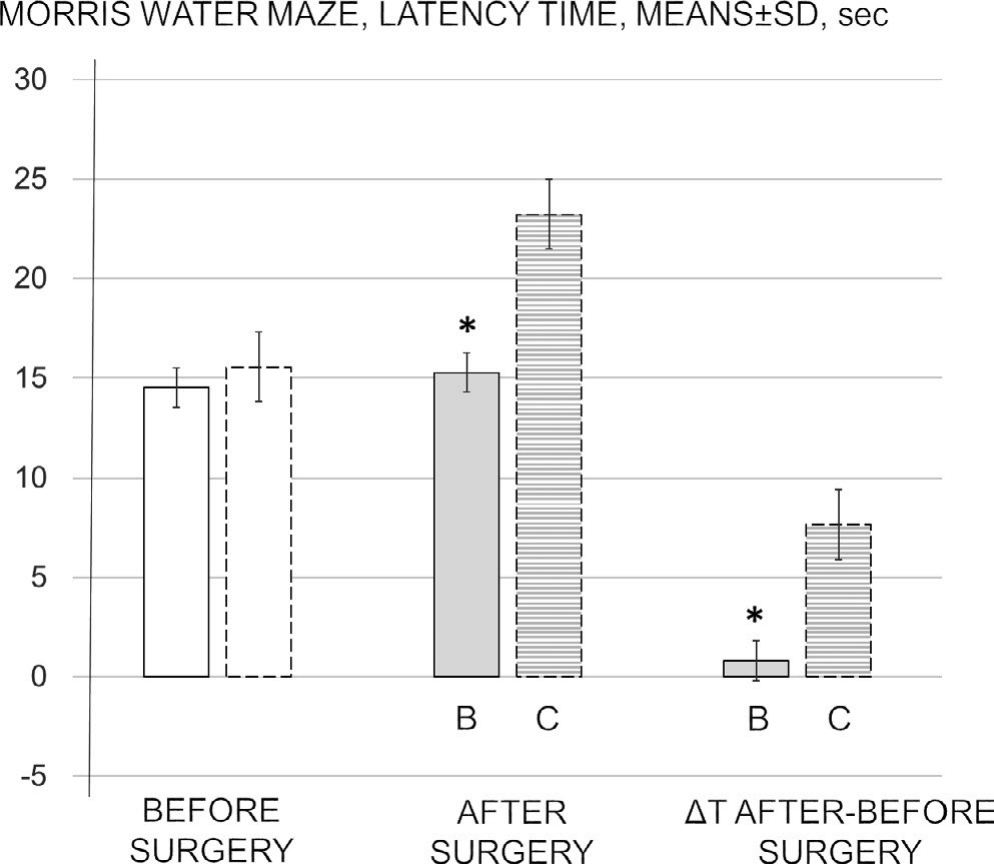
Morris water maze, latency time, means ± *SD*, sec. The day five latency time taken as a baseline measure before surgery (white bars). The effect of medication during reperfusion (gray bars) BPC 157 (b, full bars) or saline (control, c, dashed bars) assessed at 24 hr of reperfusion after surgery. The difference between training latency time and postoperative latency time as ΔT (after‐before surgery). *p ˂ .05 at least versus corresponding control (c)

Morris water maze test. After surgery, latency time markedly increased in all control rats at 24 hr of reperfusion. BPC 157 completely counteracted the ischemia/reperfusion‐induced damage (Figure 1). Namely, rats that received BPC 157 during reperfusion maintained their initial latency time from before surgery.

**FIGURE 2:**
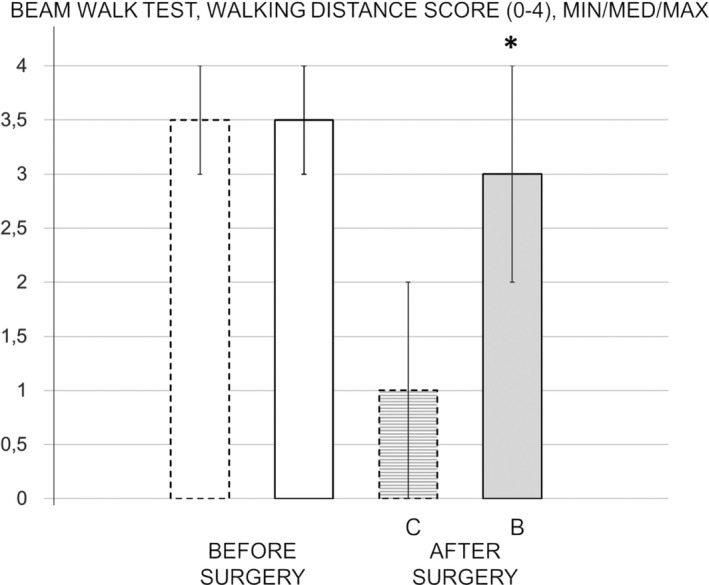
Inclined beam walk test, scored 0–4, Min/Med/Max. Presentation before surgery taken as a baseline measure (white bars). The effect of medication during reperfusion (gray bars), BPC 157 (B, full bars), or saline (control, C, dashed bars) assessed at 24 hr of reperfusion after surgery. *p ˂ .05 at least versus corresponding control (C)

Inclined beam walk test. All control rats at 24 hr of reperfusion exhibited the complete lack of fore and hind limb motor coordination and the inability to walk a short distance (Figure 2). Contrarily, this ischemia/reperfusion‐induced defect did not appear after the BPC 157 administration. Namely, rats that received BPC 157 during reperfusion were able to walk the whole distance.

**FIGURE 3:**
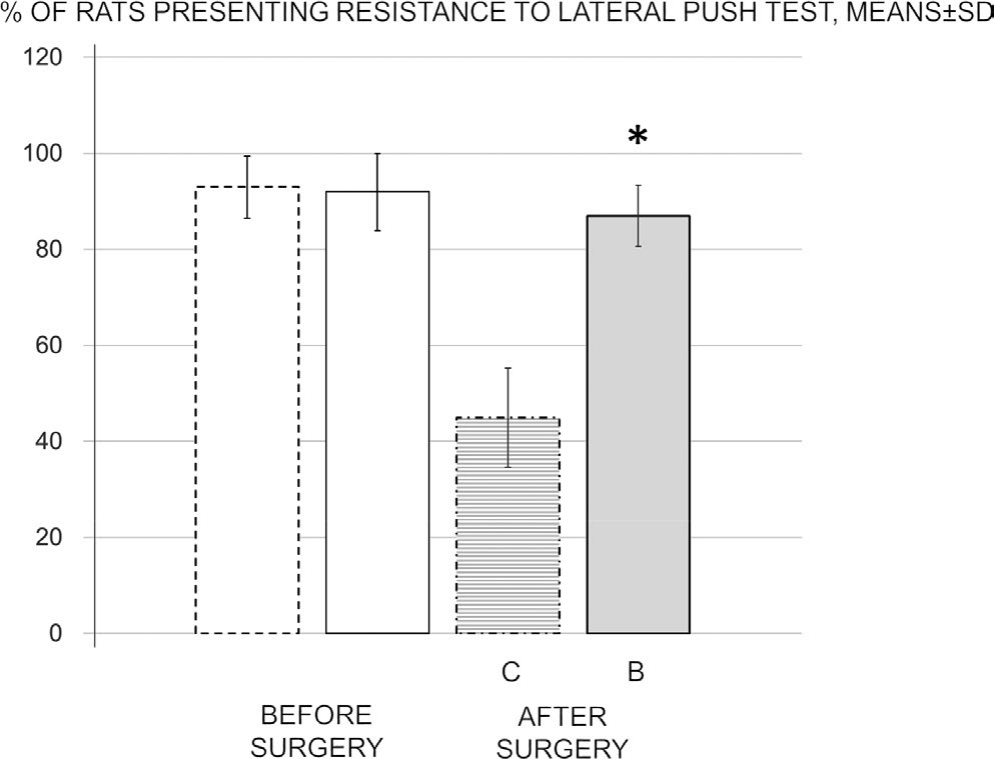
Percentage of rats presenting resistance to lateral push from either side of the shoulder, means ± *SD*. Presentation before surgery taken as a baseline measure (white bars). The effect of medication during reperfusion (gray bars), BPC 157 (B, full bars), or saline (control, C, dashed bars) assessed at 24 hr of reperfusion after surgery. *p ˂ .05 at least versus corresponding control (C)

Lateral push test. At 24 hr of reperfusion in stroke rats, BPC 157‐treated rats maintained an unimpaired ability to resist lateral pushes (Figure 3), unlike the controls, which all lacked resistance to lateral pushes from either side of the shoulder.

### 3.2 Effect of BPC 157 on brain pathology

**FIGURE 4:**
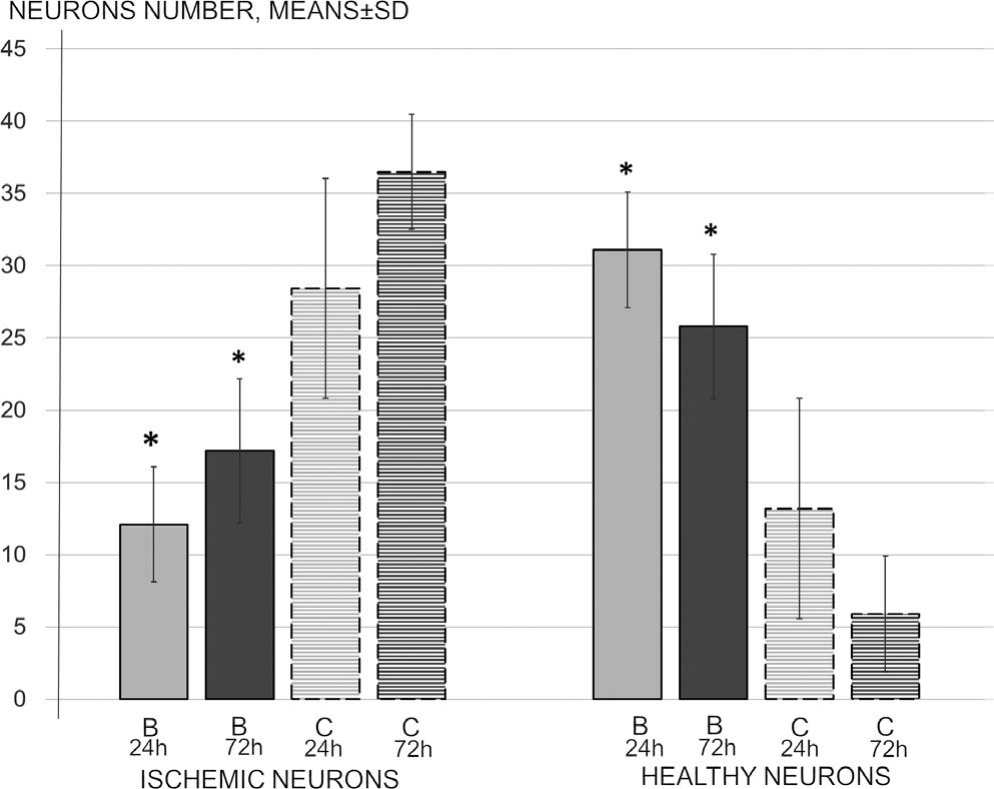
Neurons number, ischemic and healthy neurons at 24 and 72 hr reperfusion time, means ± *SD*. The effect of medication during reperfusion (gray bars), BPC 157 (B, full bars), or saline (control, C, dashed bars) assessed at 24 hr (light gray) and 48 hr (dark gray) of reperfusion after surgery. *p ˂ .05 at least versus corresponding control (C)

**FIGURE 5:**
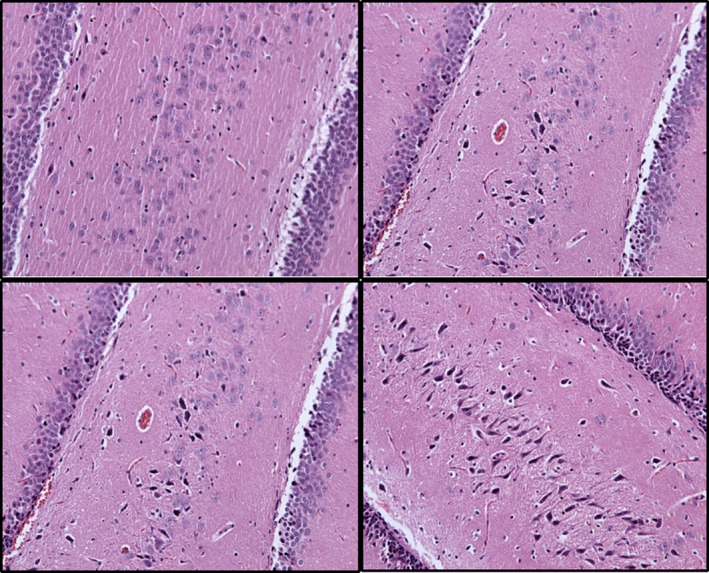
Hippocampus (CA1‐4 region) ischemic and healthy neurons presentation (HE, ×200). Images were taken at 24 hr (upper) and 72 hr (lower) of reperfusion after administration of medication. BPC 157 (left) or saline (control, right) at 24 hr (upper) and 72 hr (lower) of reperfusion after surgery

By manually counting all “red neurons,” which are those that are pathologically altered due to ischemia and reperfusion injuries, as well as healthy neurons at 24 and 72 hr of reperfusion, we revealed that BPC 157‐treated animals had far less neuronal damage than control animals and consistently had more healthy neurons (Figures 4 and 5). Thus, BPC 157 therapy seems to counteract delayed neuronal death, which consistently appears in the control of ischemia/reperfusion rats.

### 3.3 RT‐qPCR mRNA measurement

**FIGURE 6:**
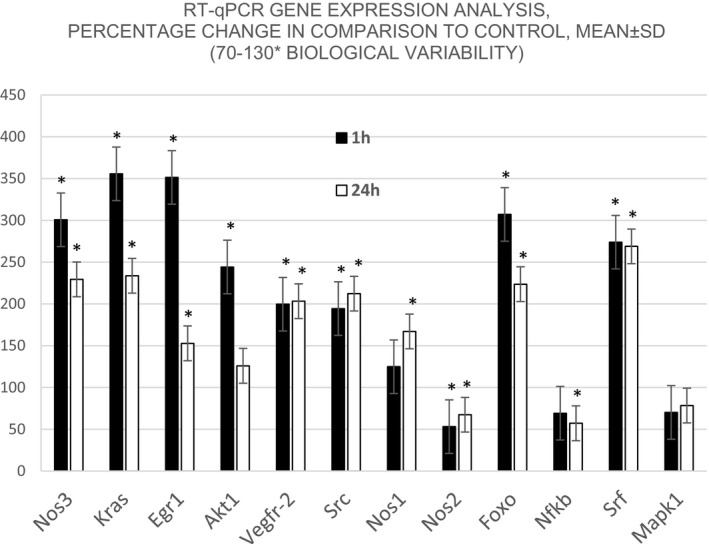
RT‐qPCR changes in mRNA levels in the hippocampus (CA1‐4 regions) expressed as percentages (mean ± *SD*, * marks significant value (*p* ≤ .05), in comparison with control animals. Selected genes were tested in two‐time intervals, 1 hr (black bars) and 24 hr (white bars), respectively. Values in the range between 70% and 130% are considered biological variability, while those under that range are under‐regulated and those above that range are upregulated

In rats with stroke‐reperfusion at 1 and 24 hr, the beneficial effect of BPC 157 therapy during reperfusion was associated with strongly elevated (*Egr‐1, Akt‐1, Kras, Src, Foxo, Srf, Vegfr2, Nos1,* and *Nos3*) and decreased (*Nos2* and *Nfkb1*) gene expression, while *Mapk1* remained unchanged throughout both time intervals (Figure 6).

In conclusion, we demonstrated that BPC 157 application during reperfusion has a neuroprotective strategy that reduces ischemic neuronal damage and attenuates or even completely prevents ischemia/reperfusion‐induced behavioral deficits in the water maze test and motor coordination failure in the inclined beam walk and lateral push test. These results are accompanied by a consistent effect on the mRNA expression of several genes (*Vegfr2, Src, Nos2, Nos1, Nos3, Akt1, Kras, Mapk1, Srf, Foxo1, Nfkb1,* and *Egr1*).

## 4 DISCUSSION

With bilateral carotid artery ligation and reversal, this ischemia/reperfusion study reflects the full extent of stroke injuries in rats more than previous drugs studies have (Danduga, Reddy, Seshadri, Has, & Kumar, 2018; Sobrado, Lopez, Carceller, Garcia, & Roda, 2003; Yang, Pan, Chen, Cheng, & Wang, 2007). Namely, previous studies consistently used significant preconditioning to avoid specific application during reperfusion (Danduga et al., 2018; Sobrado et al., 2003; Yang et al., 2007). Unlike previous preconditioning ischemia/reperfusion studies (Danduga et al., 2018; Sobrado et al., 2003; Yang et al., 2007) (i.e., attenuated ischemia to attenuate reperfusion), the new approach of BPC 157 application during reperfusion, as in the ischemic/reperfusion ulcerative colitis study (Duzel et al., 2017), directly resolves reperfusion, immediately acting specifically on the ongoing reperfusion cascade in the damaged brain. As an advantage of this stroke study after ischemia, this further ascertains the stable gastric pentadecapeptide BPC 157 effect (Kang et al., 2018; Seiwerth et al., 2014, 2018; Sikiric et al., 2010, 2011, 2012, 2013, 2014, 2016, 2017, 2018) as reperfusion therapy. We emphasize its combined effect on hippocampal neurons, which are known to be particularly vulnerable (Iwasaki et al., 2003), counteracting both early and delayed neural damage and achieving full functional recovery (based on the Morris water maze test, inclined beam‐walking test, and lateral push test). After the removal of the bilateral carotid artery ligation, considering the large extent of the behavioral and learning disturbances in these models, we found that functions were generally maintained after BPC 157 treatment. Notably, severely impaired locomotion capabilities including a lack of fore and hind limb motor coordination and resistance to lateral pushes from either side of the shoulder, are regular in control stroke rats (Gulati, Singh, & Muthuraman, 2014). Likewise, the Morris water maze test, which strongly correlates with hippocampal synaptic plasticity and NMDA receptor function (Vorhees & Williams, 2006), involves the entorhinal and perirhinal cortices, the prefrontal cortex, the cingulate cortex, the neostriatum and perhaps even the cerebellum in a more limited way (Vorhees & Williams, 2006). This is something that has already been described for BPC 157 therapy and agrees with the counteraction of various encephalopathies (Drmic et al., 2017; Ilic et al., 2009, 2010; Ilic, Drmic, Franjic, et al., 2011; Ilic, Drmic, Zarkovic, et al., 2011; Iwasaki et al., 2003; Klicek et al., 2013; Lojo et al., 2016), attenuation of concussive brain injury (Tudor et al., 2010), and maintained function, that is, preserved consciousness and fewer seizures (Drmic et al., 2017; Ilic et al., 2009, 2010; Ilic, Drmic, Franjic, et al., 2011; Ilic, Drmic, Zarkovic, et al., 2011; Iwasaki et al., 2003; Klicek et al., 2013; Lojo et al., 2016; Tudor et al., 2010) or less severe behavioral disturbances (Jelovac et al., 1998, 1999; Sikiric et al., 2002, 2016). Additionally, this would be similar to its effect on occluded vessels, as it bypasses occlusion and reestablishes blood flow (Amic et al., 2018; Drmic et al., 2018; Duzel et al., 2017; Sever et al., 2019; Vukojevic et al., 2018). The supportive analogy agrees with the successful ischemia/reperfusion therapy demonstrated in the rats with the infrarenal occlusion of the inferior caval vein, ischemic/reperfusion colitis, duodenal congestion and cecum perforation injuries (Amic et al., 2018; Drmic et al., 2018; Duzel et al., 2017; Sever et al., 2019; Vukojevic et al., 2018) and those with bile duct induced liver cirrhosis and portal hypertension (Sever et al., 2019). The vessel recruitment (described as bypassing pathways arising from different vessel tributaries to establish blood flow continuity) in these rats (Amic et al., 2018; Drmic et al., 2018; Duzel et al., 2017; Sever et al., 2019; Vukojevic et al., 2018) was due to BPC 157 therapy. Further consequences of BPC 157 therapy (i.e., the prevention and reversal of both caval hypertension and aortal hypotension; the counteraction of tachycardia, thrombosis, and thrombocytopenia; and the amelioration of consequently prolonged bleeding) provides ample evidence that the Virchow triad was abolished (Vukojevic et al., 2018). Likewise, there is preserved and rescued intestinal mucosal integrity and vein integrity, reversed portal hypertension along with recovery of bile duct ligation‐induced cirrhosis in rats (Sever et al., 2019), and reduced or even normal MDA levels in both ischemic and reperfusion conditions in various tissues (i.e., colon, duodenum, cecum, liver, and veins) and plasma (Amic et al., 2018; Drmic et al., 2018; Duzel et al., 2017; Sever et al., 2019; Vukojevic et al., 2018). Furthermore, in rats with an infrarenally occluded inferior caval vein (Vukojevic et al., 2018), we observed a particular expression pattern of the essential factors that modulate many genes and processes such as diverse pro‐adhesive, proinflammatory, and pro‐thrombotic genes upon vascular injury (Ansari, Ansari, Andersson, & Andren‐Sandberg, 2015; Hinterseher et al., 2015; Schweighofer, Schultes, Pomyje, & Hofer, 2007), *Egr1, Nos3, Srf, Vegfr2, Akt1, Plcɣ,* and *Kras* (Vukojevic et al., 2018). It was suggested that they showed a particular venous expression, distinctive between the pathways that were occluded (inferior caval vein), appeared as bypassing pathway (left ovarian vein), or blinded pathway (right ovarian vein) (Vukojevic et al., 2018). Of note, similar to the situation in rats with inferior caval vein occlusion (Vukojevic et al., 2018), RT‐qPCR has the limitation of results only reflecting mRNA levels, which may not correlate with protein levels. Nevertheless, the mRNA expression analyses provide insight into events in the rats after removal of the bilateral carotid artery ligation. They are similar to that seen before in the rats with the occlusion of the inferior caval vein, which demonstrated the left ovarian vein and other veins recruitment to bypass occlusion of the inferior caval vein (Vukojevic et al., 2018).

Thus, the events in the rats after removal of the bilateral carotid artery ligation may be the follow‐up of the observed activation of particular pathways with local and systemic relevance in the rats with ligation of the inferior caval vein receiving BPC 157 therapy (Vukojevic et al., 2018). These beneficial effects manifested with altered gene expression, including genes that increased (*Egr, Nos3, Srf,* and *Kras*) and genes that decreased (*Egr1, Vegfr2,* and *Plcɣ*), while *Akt1* remained unchanged in the inferior caval vein, the right ovarian vein and the left ovarian vein (Vukojevic et al., 2018). Consequently, BPC 157 efficacy in rats after stroke is proof‐of‐principle that after removal of bilateral carotid artery ligation, BPC 157 fulfills an ischemia/reperfusion stroke therapeutic effect by impacting specific pathways. Therefore, the consistent therapeutic effect of BPC 157 is accompanied by strongly elevated (*Egr1, Akt1, Kras, Src, Foxo, Srf, Vegfr2, Nos3,* and *Nos1*) and decreased (*Nos2* and *Nfkb*) gene expression, while *Mapk1* is not activated (similar to Akt1 in ICV‐rats). This may be a particular way by which the BPC 157 application may counteract the progression of reperfusion injury in the rat brain. Such pleiotropic effects agree with BPC 157 functioning through activation of several receptors (i.e., *Vegfr2* (Hsieh et al., 2017), growth hormone (Chang et al., 2014)), and interaction with several molecular pathways (Chang et al., 2011, 2014; Hsieh et al., 2017; Huang et al., 2015; Tkalcevic et al., 2007; Vukojevic et al., 2018). Illustratively, BPC 157 counteracted the increase of proinflammatory cytokines such as IL‐6 and TNF‐α (Kang et al., 2018), tumor cachexia (Kang et al., 2018), muscle wasting and changes in the expression of *Foxo3, Akt1, Mtor,* and *Gsk3b* (Kang et al., 2018).

Thereby, with the beneficial effect in rats with either inferior caval vein occlusion (Vukojevic et al., 2018) or bilateral carotid artery occlusion, there is increased *Egr1* expression, which is relevant for both, but especially so in the ischemia/reperfusion stroke rats. This likely accommodates the essential role of Egr1 indirectly controlling the expression of other genes, and thereby, neural activity, neural plasticity, and learning (Knapska & Kaczmarek, 2004), similar to the implied role of *Egr1* gene in inflammation, myocardial injury (Rayner et al., 2013), brain injuries following transient focal ischemia (Tureyen, Brooks, Bowen, Svaren, & Vemuganti, 2008) or permanent occlusion of the middle cerebral artery (Beck, Semisch, Culmsee, Plesnila, & Hatzopoulos, 2008). As a common resolving point, BPC 157 is known to simultaneously induce the expression of *Egr1* and its corepressor *Nab2* (Tkalcevic et al., 2007). *Nab2* expression is known to be regulated by some of the stimuli that also induce *Egr1* expression (Svaren et al., 1996). Thus, BPC 157, along with *Nab2*, could serve as a controlling feedback mechanism and guarantee transient and controlled *Egr1* activity (Tkalcevic et al., 2007) (consequently, *Egr1* decreases later in the 24 hr period in the inferior caval vein while remaining increased in ovarian veins (Vukojevic et al., 2018), similar to its presentation in the hippocampus of ischemia/reperfusion rats). This regulatory role of BPC 157 (BPC 157/*Egr1*) may be related to other BPC 157 interactions including BPC 157/*Akt1* (elevated *Akt1* expression in stroke rats, but unchanged expression in inferior caval vein ligated‐rats (Vukojevic et al., 2018)), BPC 157/*Kras*, BPC 157/*Src*, BPC 157/*Foxo*, BPC 157/*Srf*, BPC 157/*Vegfr2*, BPC 157/*Nos3*, and BPC 157/*Nos1.*

Activation of *Akt1* is usually cytoprotective, such as its function during endothelial cell hypoxia (Somanath, Razorenova, Chen, & Byzova, 2006), and *Akt1* has been extensively studied and is considered to be neuroprotective in stroke (Zhao, Sapolsky, & Steinberg, 2006), with *Akt1* and *Akt3* proteins degrading as early as 1 hr after stroke (Xie et al., 2013). *Kras* function implies an intervening capillary network, preventing high‐pressure arterial blood from feeding arteries to shunt directly into the venous outflow system (Nikolaev et al., 2018). *Src* appears to be a critical component of NGF’s function—neurite growth (Kremer et al., 1991). Src interacts directly with the NMDA receptor via its unique domain (Thomas & Brugge, 1997; Yu et al., 1996). *FoxO* protein activation may be required for neuronal protection (Maiese, 2015), and a fine balance in *FoxO* activity may be required to target cognitive loss (Maiese, 2015). The upregulation of *Srf* directly controls the immediate early gene response and is an essential regulator of neuronal‐activity‐induced gene expression (Knoll & Nordheim, 2009). Of note, most of the direct neuronal effects of VEGF‐A—such as neuronal survival in cell culture models of stroke involving oxygen and glucose deprivation (Maiese, 2015) or excitotoxicity (Matsuzaki et al., 2001), hypoxic preconditioning in vitro (Wick et al., 2002), and protection in MCA occlusion models of stroke in vivo (Hayashi, Abe, & Itoyama, 1998)—have been ascribed to activation of *Vegfr2* (Farokhi‐Sisakht, Farhoudi, Sadigh‐Eteghad, Mahmoudi, & Mohaddes, 2019; Greenberg & Jin, 2013; Moriyama, Takagi, Hashimura, Itokawa, & Tanonaka, 2013).

Collectively, they may be responsible for the new balance achieved in the ischemia/reperfusion rats after BPC 157 application during reperfusion and then sustainably maintained. Illustratively, BPC 157 may also increase *Vegfr2* expression in the recovery of ischemia/reperfusion rats as a particular therapeutic effect. Namely, BPC 157 may increase and decrease and thereby control, VEGF expression during wound healing— for example, healing of a detached or transected tendon or muscle (Brcic et al., 2009)—and BPC 157 may have a prominent angiogenic effect during healing (Brcic et al., 2009; Hsieh et al., 2017; Huang et al., 2015; Sikiric et al., 1999) while counteracting the tumor‐promoting the effect of VEGF (Radeljak, Seiwerth, & Sikiric, 2004). This may be important since BPC 157 itself not only increased *Vegfr2* expression in vascular endothelial cells but also immediately triggered the internalization of *Vegfr2* and subsequent phosphorylation of *Vegfr2*, Akt, and *Nos3* signaling pathways without other known ligands or shear stress (Hsieh et al., 2017). In addition, the *Akt1*/*Nos3* signaling pathway is thought to be highly beneficial to stroke outcome, weather by upregulation of *Akt1* or by subsequent activation of *Nos3* (Zhou et al., 2015).

In support, BPC 157 largely interacts with the NO system in different models and species (Sikiric et al., 2014). Indicatively, BPC 157 alone may induce the release of NO in vitro in gastric mucosa from rat stomach tissue homogenates and counteract the opposite adverse effect of L‐NAME (i.e., hypertension; lack of NO release in vitro) and L‐arginine (i.e., hypotension; NO over‐release in vitro) (Sikiric et al., 1997). In these terms, we should consider the documented triple effect of BPC 157 on NOS in the hippocampus of ischemia/reperfusion rats, which includes the increased expression of *Nos3*, increased expression of *Nos1*, and decreased expression of *Nos2*. NO released by *Nos3* scavenges oxygen free radicals, inhibits the expression of adhesion molecules, and promotes platelet aggregation and lymphocyte adhesion (Hossain, Qadri, & Liu, 2012; Kuhlencordt et al., 2004; Moore, Sanz‐Rosa, & Emerson, 2011; Nabah et al., 2005). Inhibition of *Nos1* also produces oxygen free radicals (Gursoy‐Ozdemir, Can, & Dalkara, 2004). In contrast, the production of NO‐induced by *Nos2* leads to brain damage during ischemia/reperfusion (Gursoy‐Ozdemir et al., 2004). Overexpression of *Nos2* promotes the secretion of tumor necrosis factor‐α (TNF‐α) and interleukin‐1β (IL‐1β) and subsequently induces a secondary inflammatory reaction and the generation of oxygen free radicals (Foncea, Carvajal, Almarza, & Leighton, 2000; Trickler, Mayhan, & Miller, 2005). Thereby, the *Nos3‐Nos1‐Nos2* interplay after BPC 157 influences *Nos3* may enhance *Nos3* activity in functions such as regulating cerebral microvascular tone, protecting the blood–brain barrier, reducing oxidative stress, and alleviating procoagulant stimulation (Chen, Mou, Feng, Wang, & Chen, 2017). Of note, both the reduction of oxidative stress (Amic et al., 2018; Drmic et al., 2018; Duzel et al., 2017; Luetic et al., 2017; Sever et al., 2019; Sikiric et al., 1997; Vukojevic et al., 2018) and alleviation of procoagulant stimulation (Hrelec et al., 2009; Stupnisek et al., 2012, 2015; Vukojevic et al., 2018) have been ascribed to BPC 157 therapy. In support, we demonstrated that BPC 157 administration consistently decreased expression of *Nfkb1*, as shown previously (Huang et al., 2015), which is considered a main activator of *Nos2*, and both factors contribute to neuronal death (Liu et al., 2015). Otherwise, *Nfkb1*, as the central regulator of neuroinflammation‐associated disease pathogenesis (Niranjan, 2013), induces reactive oxygen species and proinflammatory cytokines (such as IL‐1β, interferon‐γ, and TNF‐α) that cause secondary neurotoxicity (Block, Zecca, & Hong, 2007; Kaltschmidt, Kaltschmidt, & Baeuerle, 1993) and exacerbate inflammation‐induced neurodegeneration (Yakovleva, Bazov, Watanabe, Hauser, & Bakalkin, 2011). Finally, the increased (the present ischemia/reperfusion study) or decreased (inferior caval vein occlusion study) (Vukojevic et al., 2018) expression of *Vegfr2* that coincides with continuously increased *Nos3* expression may reflect particularities of the organization of the response in the hippocampus after bilateral carotid artery ligation during reperfusion and ischemia in rats with occluded inferior caval veins (Vukojevic et al., 2018). As a final point, a notion regarding the novel ways of administering drugs (Amani, Habibey, et al., 2019; Amani, Habibey, et al., 2019), is that BPC 157 needs no carrier or peptidase inhibitor and shows no immune response or adverse reaction when applied.

## 5 CONCLUSION

Thus, compensatory pathways must exist in the cell to ensure that a critical process such as reversal of ischemia/reperfusion injuries occurs when BPC 157 is applied. With respect to the cryoprotection theory and brain‐gut and gut‐brain activity protection (Kang et al., 2018; Seiwerth et al., 2014, 2018; Sikiric et al., 2010, 2011, 2012, 2013, 2014, 2016, 2017, 2018), further studies should explore the relevance of BPC 157, as it increased the survival of cultured enteric neurons and proliferation of cultured EGCs but did not influence the proliferation of cultured enteric glial cells (Wang et al., 2019), which may improve healing of damaged enteric nervous and mucosal structures. Finally, with respect to the initial cytoprotection theory, which includes epithelium and endothelium (cyto)protection and should be further expanded to protection of other organs (Kang et al., 2018; Seiwerth et al., 2014, 2018; Sikiric et al., 2010, 2011, 2012, 2013, 2014, 2016, 2017, 2018) including the bilateral carotid artery‐ligation sequels, there is still conclusive evidence that these beneficial BPC 157 effects, including innate rapid endothelium protection, that were shown in original studies (Kang et al., 2018; Seiwerth et al., 2014, 2018; Sikiric et al., 2010, 2011, 2012, 2013, 2014, 2016, 2017, 2018) may enable stroke therapy.

## Data Availability

The data that support the findings of this study are available from the corresponding author upon reasonable request.
